# Conservation of Pollinators in Traditional Agricultural Landscapes – New Challenges in Transylvania (Romania) Posed by EU Accession and Recommendations for Future Research

**DOI:** 10.1371/journal.pone.0151650

**Published:** 2016-06-10

**Authors:** Anikó Kovács-Hostyánszki, Rita Földesi, Edina Mózes, Ádám Szirák, Joern Fischer, Jan Hanspach, András Báldi

**Affiliations:** 1 MTA Centre for Ecological Research, Institute of Ecology and Botany, Lendület Ecosystem Services Research Group, Vácrátót, Hungary; 2 Faculty of Sustainability, Leuphana University, Lüneburg, Germany; University of Roehampton, UNITED KINGDOM

## Abstract

Farmland biodiversity is strongly declining in most of Western Europe, but still survives in traditional low intensity agricultural landscapes in Central and Eastern Europe. Accession to the EU however intensifies agriculture, which leads to the vanishing of traditional farming. Our aim was to describe the pollinator assemblages of the last remnants of these landscapes, thus set the baseline of sustainable farming for pollination, and to highlight potential measures of conservation. In these traditional farmlands in the Transylvanian Basin, Romania (EU accession in 2007), we studied the major pollinator groups—wild bees, hoverflies and butterflies. Landscape scale effects of semi-natural habitats, land cover diversity, the effects of heterogeneity and woody vegetation cover and on-site flower resources were tested on pollinator communities in traditionally managed arable fields and grasslands. Our results showed: (i) semi-natural habitats at the landscape scale have a positive effect on most pollinators, especially in the case of low heterogeneity of the direct vicinity of the studied sites; (ii) both arable fields and grasslands hold abundant flower resources, thus both land use types are important in sustaining pollinator communities; (iii) thus, pollinator conservation can rely even on arable fields under traditional management regime. This has an indirect message that the tiny flower margins around large intensive fields in west Europe can be insufficient conservation measures to restore pollinator communities at the landscape scale, as this is still far the baseline of necessary flower resources. This hypothesis needs further study, which includes more traditional landscapes providing baseline, and exploration of other factors behind the lower than baseline level biodiversity values of fields under agri-environmental schemes (AES).

## Introduction

Wild pollinator populations are one of the major victims of intensive agricultural management, which diminishes available foraging resources, nesting and overwintering habitats [1]. The ecosystem service that pollinators provide is, however, crucial. Eighty-eight percent of the dicotyledonous plant species require animal pollination and the global economic value of pollination is estimated to be € 150 billion per year [2,3]. Although pollination by honey bees (*Apis mellifera* L.) has great ecological and economic value, wild pollinators, especially wild bees (Hymenotera: Apoidea) and hoverflies (Diptera: Syrphidae), play crucial roles in the pollination of several crop and wild plant species [4,5].

The Intergovernmental Platform for Biodiversity and Ecosystem Services (IPBES) dedicates special attention to the thematic assessment of pollinators, pollination and food production, reviewing the diversity, status, and trends of pollinators and pollination systems and their role in human well-being and biodiversity maintenance [6,7]. For such a comprehensive assessment, availability of data is essential; yet, the most biodiverse regions are often less-known by science [8]. Such knowledge imbalance exists at European scale as well, where the majority of available knowledge of wild pollinators is from Northern and Western Europe (i.e. the old member states of the European Union (EU) and Switzerland). However, there is currently increasing attention on Central and Eastern European (CEE) biodiversity, which is still considerably richer than in Western and Northern Europe, and may represent important baselines for conservation targets for whole Europe [9–13].

The rich biodiversity in the CEE countries is strongly linked to their agricultural production systems [12]. Large—although rapidly disappearing—extensive and/or traditionally managed areas can be found in the former socialist countries of CEE [14]. Traditional land use systems such as low-intensity livestock systems, arable and permanent crop systems, and mixed systems, persisted in Europe mainly in upland and remote areas [15]. During the second part of the 20^th^ century, however, the national and international policies, including production-related subsides of the EU’s Common Agricultural Policy (CAP) and collectivisation of farming in CEE, low-cost artificial fertilisers and pesticides forced considerable agricultural intensification, destroying or altering most of these traditionally managed agricultural ecosystems [16,17]. High-input arable farming and grassland management, increased use of pesticides, fertilisers, machinery, and loss of semi-natural habitats resulted in widespread and fast biodiversity decline [1, 18,19,20].

In the CEE countries, the use of chemicals and inorganic fertilisers increased and reached levels similar to those in the EU during the 1980s [18,21,22]. However, after the collapse of communism in the ‘90s there was a sudden decrease in state support for agriculture, resulting in land abandonment and/or declining levels of chemical use and mechanisation [18]. Despite these dramatic changes, some remote regions with resilient socio-economic systems were not affected [14]. In these regions, current farming practices are strikingly similar to those of one hundred years ago, making them a natural laboratory for studying the sustainability and conservation value of traditional agricultural ecosystems.

During the last ten years, however, 11 former socialist countries joined the EU. Membership opened new EU sources under the Common Agricultural Policy (CAP), primarily to support management intensification. This threatens the high diversity of low-intensity agricultural landscapes [23]. Both fertiliser and pesticide use are increasing in CEE [22], while biodiversity, seems to be declining [22,24]. To avoid long-term biodiversity decline that was experienced in the old member states of the EU in the second half of the 20^th^ century [18,19], we need convincing evidence on effects of sustainable land use on patterns and functions of biodiversity. Such evidence, in turn, can act as a baseline for efficient measures and then be used to shape farmland subsidies towards regionally tailored, effective farmland management. While the CAP also introduced measures, called agri-environment schemes (AES), to subsidize “nature-friendly farming”, they are largely blind-copied from the Western European systems and may not be well-suited to the CEE countries, due to the different landscape context and management history [12,13,24].

**Fig 1:**
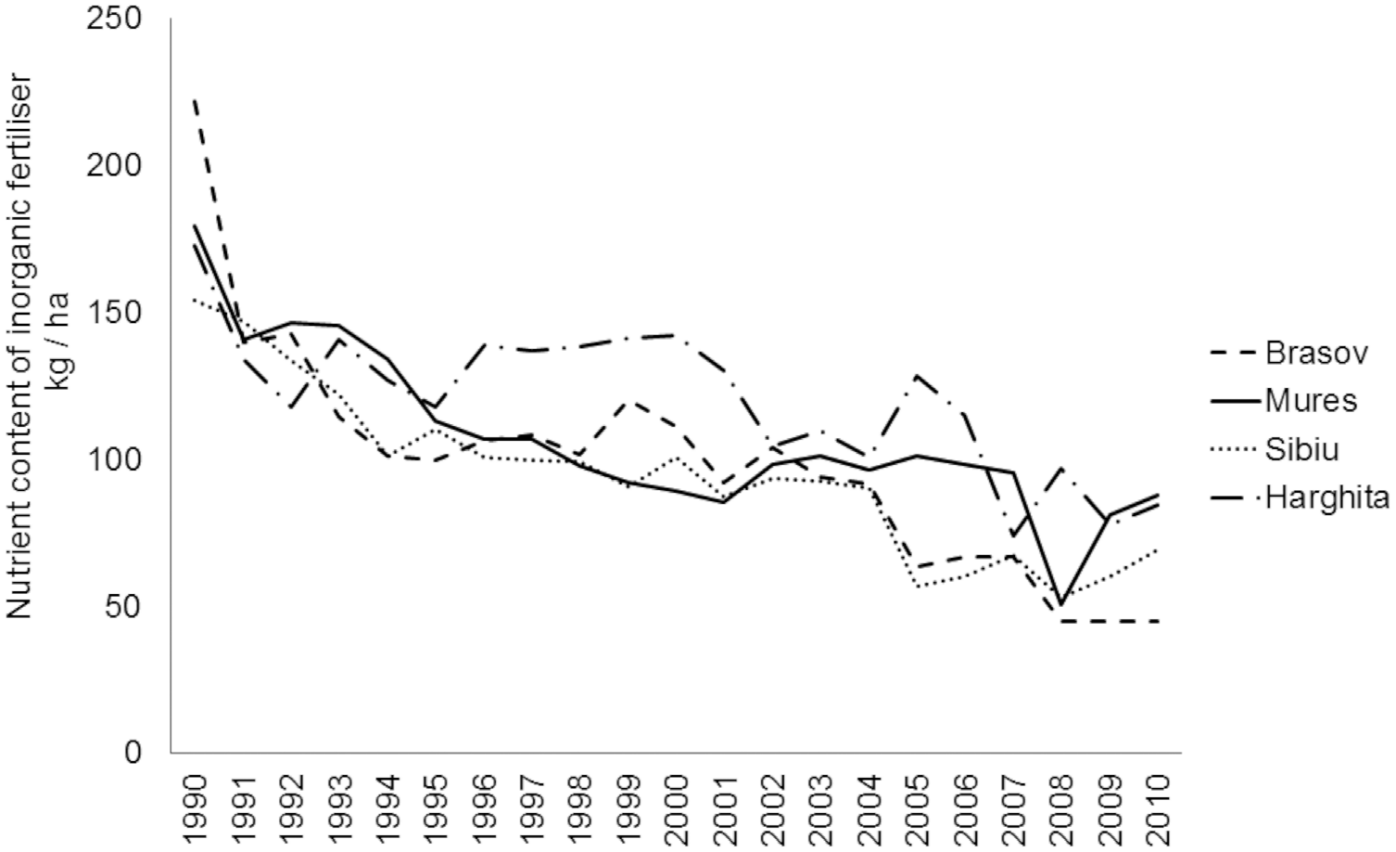
Nutrient amount of inorganic fertilisers (kg/ha) used in four counties in Southern Transylvania after the collapse of the socialist system in 1990.

**Fig 2:**
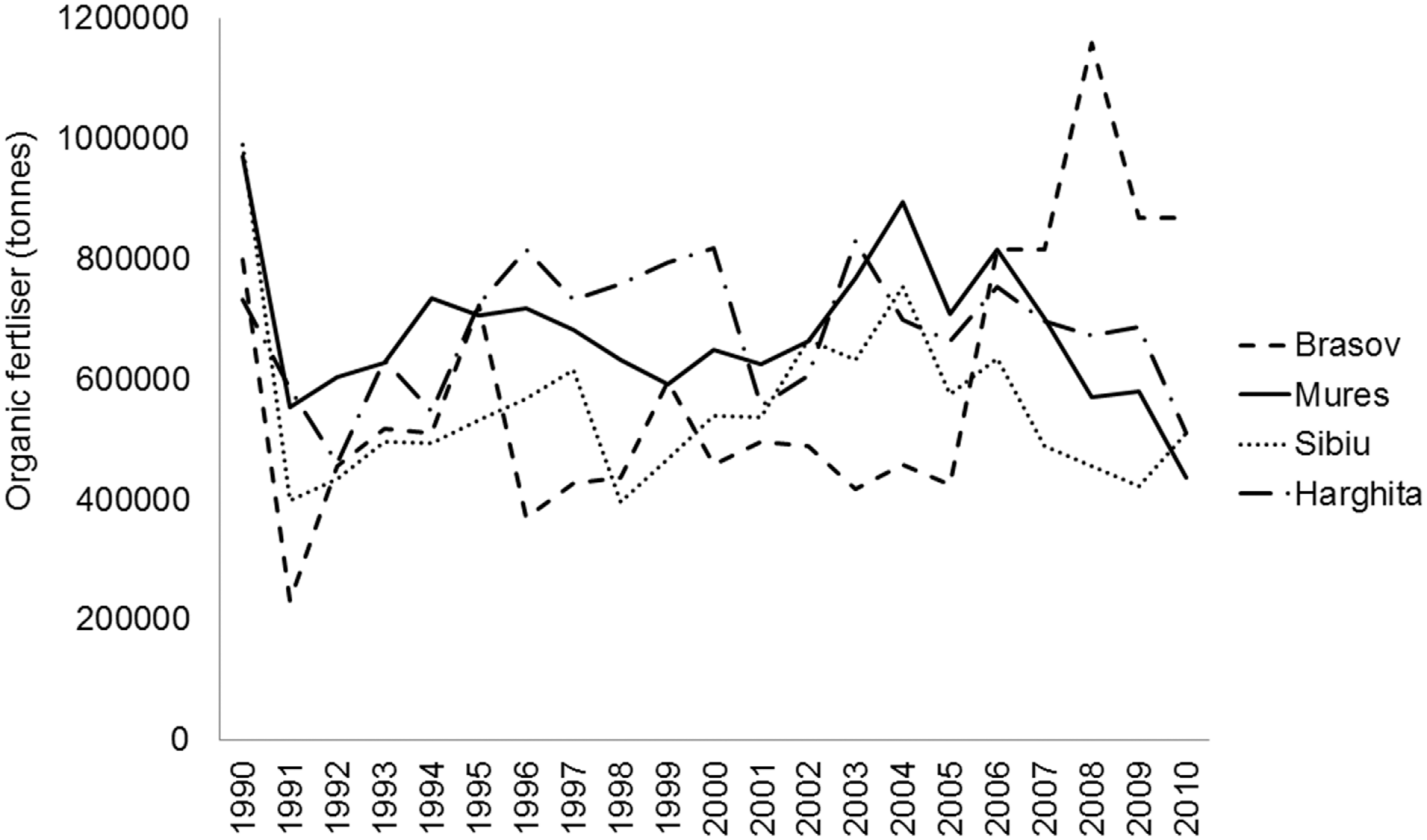
Amount of organic fertilisers (tonnes) used in four counties in Southern Transylvania after the collapse of the socialist system in 1990.

**Fig 3:**
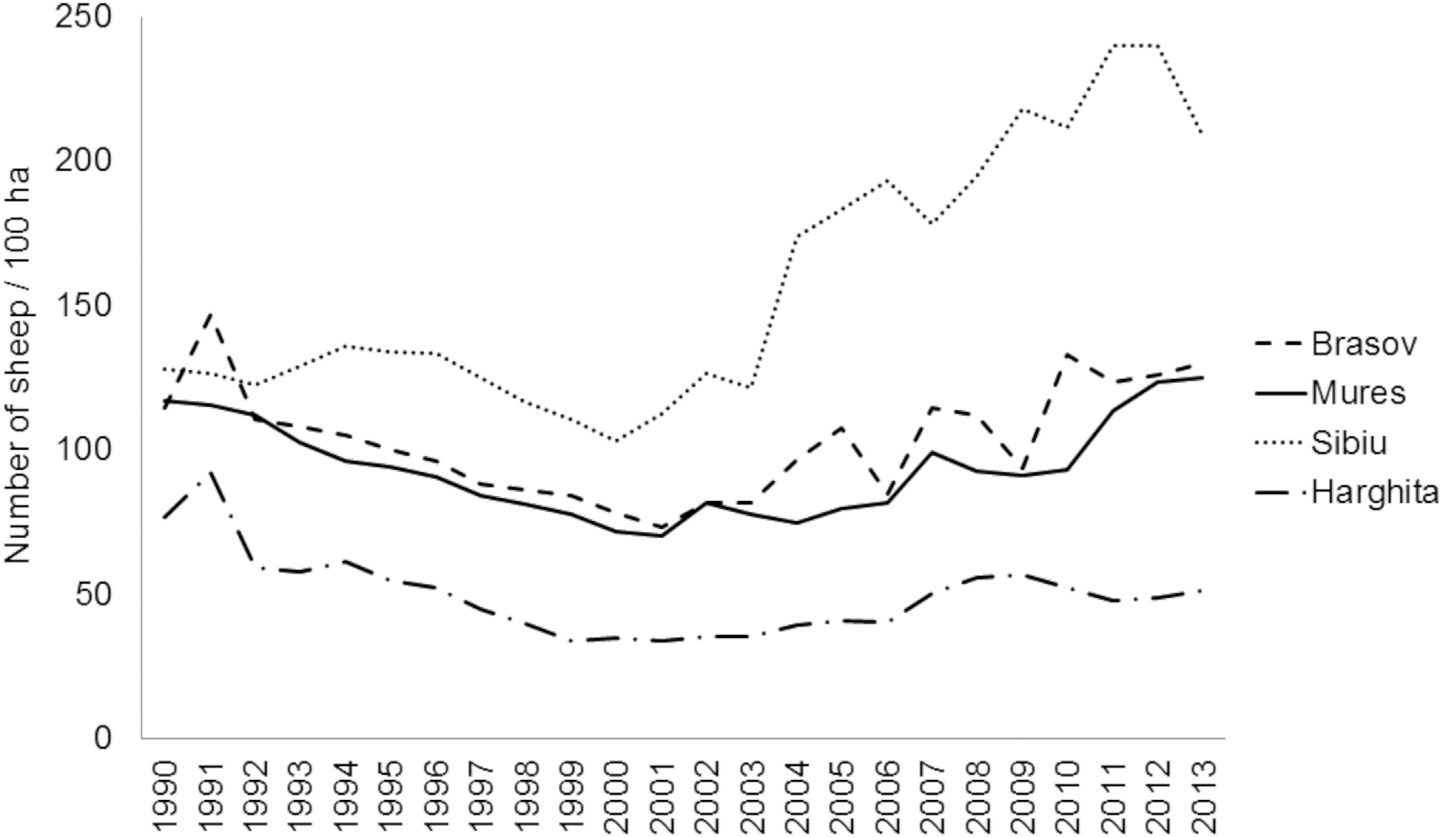
Number of sheep in four counties in Southern Transylvania after the collapse of the socialist system in 1990.

To fill some of this knowledge gap on pollinator communities in CEE and to provide baseline for conservation efforts, we studied wild bees, hoverflies and butterflies (Rhopalocera) in traditional agricultural landscapes in Southern Transylvania, Romania, where traditional land use and farmland management sustain rich, but still largely understudied, highly diverse flora and fauna [14,25]. The land use practices of local people shaped this traditional farming landscape, which is still characterised by high percentage of semi-natural vegetation and biodiversity despite the political and economic changes during the 20^th^ century [26]. After the collapse of communism in 1989, much cropland was abandoned (approximately 28% between 1990 and 2005 at Romanian scale), posing considerable risk to species depending on low-intensity agricultural lands [27]. Intensification has not yet progressed very far in the region; mechanisation and application of artificial fertilisers and pesticides are still at low levels. Mineral fertiliser application declined (Fig 1), organic fertiliser use remained high (Fig 2), while grazing by sheep and cattle declined after the collapse of the socialist system (Fig 3) [28].

Both land use and land management changes via intensification or abandonment can alter landscape heterogeneity and the amount or distribution of semi-natural landscape elements, having possible negative consequences on the pollinator communities seeking for nesting place and foraging resources within their closer and wider environment around their nests [29]. However, these effects can be different according to the different pollinator groups, dispersal ability and diet breadth [29,30]. In our study we aimed to: (1) access the effects of various scale landscape structures and local scale drivers shaping pollinator insect communities in the traditional agricultural landscapes of Southern Transylvania, Romania; (2) evaluate the conservation value of the recorded wild bee species using European Lists and the difference between areas with and without conservation interest; (3) generate hypotheses, further research questions, and bring attention to the potential consequences of certain agricultural management changes at landscape and local scales on the wild bee, hoverfly and butterfly communities. We discuss our results according to a Transylvanian example of three counties, attributed still a wide range of traditional agricultural practices applied and considerable biodiversity values.

## Materials and Methods

### Ethics statement

Permission for field surveys within the Natura 2000 network was granted through Progresul Silvic, the organization officially entrusted with custody of the protected area by the Romanian government. No specific permissions were required for other locations, since the impact on the studied environment and managed fields was minimal. The field study did not involve any endangered or protected species. One bee species was classified as endangered on the IUCN European Red Bee list, which was applied in the evaluation of the wild bee data, but was published only three years after the field survey.

### Study area

The study area was situated in Mureş, Sibiu, Braşov and Harghita counties of Southern Transylvania, Romania (see S1 Fig). The landscape in the studied region is characterised by the mosaic of small parcels of low-intensity arable fields (15% cover, horse ploughing still in use, no or low amount of artificial agrochemicals are used), pastures (40% cover, low-intensity grazing and/or mowing) in the valley and on the slopes and deciduous forests (33% cover) on the hilltops [26].

In a ca. 50 km diameter vicinity of Sighişoara (Segesvár) 19 village catchments were chosen for the study in 2012. Village catchment was chosen as the landscape unit, because it represents a tangible unit suitable to understand social-ecological interactions. Regarding landscape topography, there were 11 highly complex catchments with extensive forest, steep slopes, and small arable fields, and 8 low complexity catchments with little forest and flatter surfaces. Protection status of the catchments was either unprotected, SCI (Site of Community Importance) or SPA (Special Protection Area). Within each catchment we typically sampled two arable fields and two grasslands (land use types), yielding 38 and 38 sites respectively (see S1 Fig; for further details of study site selection see [25,31]).

The crop/vegetation structure covered a wider range of different types, which were classified according to the following categories (crops): grassland with shrubs (N = 7), pasture (grazed by cattle or sheep; N = 24), hay meadow (N = 10), fallow (N = 4), alfalfa (N = 15), cereal (winter wheat and barley; N = 8) and corn (N = 8).

### Pollinator survey

Pollinating insects were surveyed by walking along two 100 m long and 3 m wide transects (1.5 m either side) per field, running parallel, at least 30 m from the edge and 50 m from each other. Four observers walked along these transects in two pairs in one direction, sampling pollinator insects within the transect over 20 min by an insect net according to the transect walk method. Bumblebees (*Bombus* spp.), other wild bees and hoverflies were caught with an insect net, transferred into a killing jar with ethyl acetate, and identified in the laboratory [32–38]. Some specimens that we were not able to catch in the field were noted at genus level. Butterflies were only counted, but not identified at species level. Sampling was carried out three times during the season (May, June, July) in 10–12 days periods on dry and warm days with minimal wind, and 20°C minimum temperature, between 09.00 and 18.00 o'clock. Sampling on a given location alternated between morning and afternoon among the three sampling periods.

During the analyses, we distinguished between bumblebees and other wild bees because these two groups have different biological traits in terms of floral requirements, flying abilities and sociality [39–41], and therefore different responses to landscape and local scale environmental conditions were expected. Bumblebees are larger than most other bees and forage on a range of one to several kilometres, whereas many other bees forage within a few hundred metres of their nesting sites [42,43]. Although there are some semi-social species and/or genera among the collected bees (e.g. some *Halictus* spp.), we use the terminology ‘solitary bees’ in the current paper in the case of wild bees other than bumblebees.

Wild bee species were categorised by host range as oligolectic and polylectic species. Oligolecty refers to a narrow host plant spectrum, collecting pollen and/or nectar only from a restricted number of plant genera/family, while polylecty means various host plant genera belonging to at least four plant families [44].

To evaluate the conservation concern of the found bee fauna in our study, wild bee species were checked for conservation status. IUCN European Red List of Bees was used to evaluate conservation status at geographical Europe level [45]. Species were classified according to IUCN Red list categories as regionally extinct (RE), threatened (critically endangered—CR, endangered—EN, vulnerable—VU) or near threatened (NT) species.

### Environmental data (botanical survey and calculation of landscape parameters)

Environmental data were assessed at different spatial scales. At the landscape scale we quantified percentage area of arable fields and semi-natural habitats (vineyards; fruit trees and berry plantations; pastures; complex cultivation patterns; land principally occupied by agriculture, with significant areas of natural vegetation; natural grasslands; transitional woodland-shrub) and calculated Shannon index of land cover diversity (land cover categories: urban, arable, semi-natural, forest, water) within 1000 m radius circle around our sampling sites based on CORINE land cover data [46]. Variables of landscape composition were measured by ARCGIS software [47].

Two parameters were measured around the study sites within a 1 ha circle (56.42 m radius): heterogeneity and woody vegetation cover [31]. Heterogeneity was defined as the standard deviation of the 2.5m panchromatic Spot picture, stratified in quantiles: H1 –belonging to lower third (low heterogeneity), H2 –middle third, H3 –upper third. Woody vegetation cover represented the proportion of woody vegetation (shrubs and trees) in a 1 ha circle based on classified 10m SPOT 5 data: W1: 0 to 5% woody vegetation cover; W2: 5 to 15% of woody vegetation; W3: 15 to 50% of woody vegetation (for more details see also [25,31]).

At the local scale, we collected data on flowering, insect-pollinated plant species and number of their flowers at species level along the pollinator sampling transects in ten 1*1 m quadrates 10 meters apart at each pollinator sampling occasion. We calculated an index of ‘flower abundance’ per transect by taking the arithmetic average of the number of flowers of each species over the ten quadrates and over the three sampling periods. The number of flowering plant species along the transect was pooled as ‘flower species richness’ over the whole sampling.

### Statistical analyses

To calculate species richness data we pooled observed wild bee and hoverfly species from the three survey periods for each survey transects according to the followings: all wild bees, oligolectic wild bees, polylectic wild bees, wild bees of conservation interest, hoverflies. Arithmetic mean abundance over the three survey periods were calculated to the: solitary wild bees, bumblebees, oligolectic wild bees, polylectic wild bees, wild bees of conservation interest, hoverflies and butterflies.

First, we tested differences in species richness and abundance between different levels of official protection (protected (SCI or SPA) vs. non-protected) and different land use types (arable vs. grassland) by using Wilcoxon rank-test. Difference among the different crop types was tested by ANOVA, species richness and abundance values were log_10_ transformed to reach normal distribution.

Second we used generalized linear mixed effects models (GLMMs) to assess effects of different landscape and local scale predictors on the species richness and abundance of pollinators. Species richness and abundance data were log_10_ transformed to reach normal residual distribution. As variables sampled at nearby locations (sites in the neighbourhood of one village) are not independent from each other, all dependent variables were tested for spatial autocorrelation [48] using Moran’s I test in R package *ape*. The autocovariate was extracted using the function in *autocov dist* R package *spdep*. We tested all predictor variables for multicollinearity by calculating the variance inflation factor (VIF) using *vif* function of the *fmsb* package in R or Chi-squared test of independence for categorial variables. A maximum VIF value of 5 was taken as an indicator of multicollinearity [49].

As explanatory variables in the GLMMs we used as covariates percentage of semi-natural habitats and Shannon index of land cover diversity within 1000 m radius, heterogeneity and woody vegetation cover within 1ha and total flower species richness (S1 Table). Hence percentage area of semi-natural habitats strongly correlated with percentage of arable fields (Spearman correlation: R = -0.66, p < 0.001) and showed significant difference between the different levels of topography (t-test: t = 2.33, df = 87.25, p-value = 0.022), higher values of the high topography, we included only semi-natural habitat % into the analyses. We used only flower species richness in the analyses, as it showed strong correlation with flower abundance (Spearman correlation: R = 0.67, p < 0.001). Pollinator data were analysed at transect level as collected, which were nested according to study site (‘Site’) and those in village catchments (‘Village’) as random factors: village/site. In addition, all pair-wise interactions were tested between the explanatory variables. Terms were removed sequentially in backward stepwise selection until only significant interactions and main effects (P > 0.05 from F test) remained in the minimal adequate model. Analyses were performed using the *nlme* [50], *stats* [51], *multcomp* [52], and *mvtnorm* [53] packages of R statistical environment version 3.0.1 [51].

Community species composition of pollinator assemblages was studied using partial redundancy analyses (RDA). Separate analyses were conducted for bees and hoverflies in the whole sampling. The species matrices were constrained by percentage of semi-natural habitats and Shannon index of land cover diversity within 1000 m radius, heterogeneity and woody vegetation cover within 1ha and total flower species richness. Hellinger transformation was performed for each species matrix allowing the use of ordination methods such as RDA, which is Euclidean-based, with community composition data containing many zeros [54]. Calculations were performed using the *vegan* package (version 1.16, [55]).

Flower species richness was tested in the function of crop using ANOVA.

## Results

We sampled 3390 individuals of wild bees (2332 individuals of 150 species caught and identified in the laboratory, and 1058 individuals of *Bombus* and *Halictus* genera identified in the field), 1481 individuals of hoverflies (1097 individuals of 46 species caught and identified in laboratory, 384 individuals identified at family level in the field) (S2 and S3 Tables) and counted 3929 individuals of butterflies. Forty-two wild bee species were classified as oligolectic, 85 as polylectic (S2 Table). One species was endangered, two species vulnerable and 13 near threatened at the European scale (S2 Table).

Protected and unprotected sites did not differ in species richness of wild bees or hoverflies, but abundance of solitary wild bees, polylectic wild bees and butterflies was significantly higher in non-protected than in protected sites (Table 1). Grasslands had a significant higher species richness of wild bees, species richness and abundance of polylectic wild bees, and higher abundance of butterflies than arable land. Arable lands had a higher abundance of bumblebees, oligolectic wild bees and hoverflies than grasslands (Table 1). Species richness and abundance of all pollinator groups showed significant difference between certain crops (S4 Table, S2 Fig).

**Table 1:** The difference between protected (P: SCI or SPA) and non-protected (NP) sites (Protection status) and between arable fields (A) and grasslands (G) (Land use) in the species richness and abundance of the different pollinator insect groups according to the Wilcoxon rank test. P-values of significant effects are in bold.

	Protection status			Land use		
	W	p		W	p	
**Species richness**						
Wild bees	3294.5	0.074		2242	**0.017**	A<G
Oligolectic bees	3105	0.261		3230.5	0.188	
Polylectic bees	3291	0.076		2019	**0.001**	A<G
Wild bees of conservation interest	2892.5	0.744		2807	0.733	
Hoverflies	2390	0.110		3092.5	0.448	
**Abundance**						
Solitary wild bees	3576.5	**0.005**	NP>P	2399	0.072	
Bumblebees	2331	0.067		3664.5	**0.004**	A>G
Oligolectic bees	3221	0.119		3492	**0.022**	A>G
Polylectic bees	3397.5	**0.030**	NP>P	2296.5	**0.029**	A<G
Wild bees of conservation interest	2987	0.470		2816	0.765	
Hoverflies	2511	0.255		4031	**<0.001**	A>G
Butterflies	3648.5	**0.002**	NP>P	1416.5	**<0.001**	A<G

Percentage of semi-natural habitats within 1000 m had a significant positive effect on the species richness and abundance of polylectic wild bees and abundance of butterflies, but it correlated negatively with the abundance of hoverflies (Table 2). Shannon land cover diversity had no significant effects. Woody vegetation cover within 1ha area had a significant positive effect on the species richness of oligolectic wild bees and the abundance of solitary wild bees. Flower species richness showed significant positive effect on the species richness and abundance of all pollinator groups except the abundance of bumblebees, oligolectic wild bees and hoverflies, and species richness and abundance of wild bees of conservation interest.

**Table 2:** The effects of percentage of semi-natural habitats and Shannon land-cover diversity in 1000 m radius, heterogeneity and woody vegetation cover in a given 1 ha circle area, and flower species richness on the species richness and abundance of the different pollinator insect groups according to the linear mixed effect models. P-values of significant effects are in bold

	df	F	p
**Species richness**			
Wild bees			
Semi-natural %—1000 m	1, 54	2.58	0.114
Heterogeneity—1 ha	1, 54	0.55	0.461
Flower species richness	1, 75	46.44	**<0.001**
Semi-natural*Heterogeneity	1, 54	4.26	**0.044**
Oligolectic bees			
Wood cover—1 ha	1, 56	4.23	**0.044**
Flower species richness	1, 75	9.07	**0.004**
Polylectic bees			
Semi-natural %—1000 m	1, 56	6.51	**0.014**
Flower species richness	1, 75	54.41	**<0.001**
Wild bees of conservation interest			NS
Hoverflies			
Semi-natural %—1000 m	1, 54	1.20	0.279
Heterogeneity—1 ha	1, 54	0.24	0.625
Flower species richness	1, 75	9.93	**0.002**
Semi-natural*Heterogeneity	1, 54	4.40	**0.041**
**Abundance**			
Solitary wild bees			
Semi-natural %—1000 m	1, 52	2.06	0.157
Heterogeneity—1 ha	1, 52	0.50	0.481
Wood cover—1 ha	1, 52	4.58	**0.037**
Flower species richness	1, 75	37.79	**<0.001**
Semi-natural*Heterogeneity	1, 52	8.81	**0.005**
Heterogeneity*Wood cover	1, 52	4.54	**0.038**
Bumblebees			
Heterogeneity—1 ha	1, 56	0.03	0.865
Flower species richness	1, 74	1.78	0.186
Heterogeneity*Flower species richness	1, 74	7.84	**0.007**
Oligolectic bees			NS
Polylectic bees			
Semi-natural %—1000 m	1, 56	6.80	**0.012**
Flower species richness	1, 75	34.80	**<0.001**
Wild bees of conservation interest			NS
Hoverflies			
Semi-natural %—1000 m	1, 56	7.34	**0.009**
Butterflies			
Semi-natural %—1000 m	1, 56	7.57	**0.008**
Flower species richness	1, 75	62.61	**<0.001**

“*” indicates interaction between the two variables.

**Fig 4:**
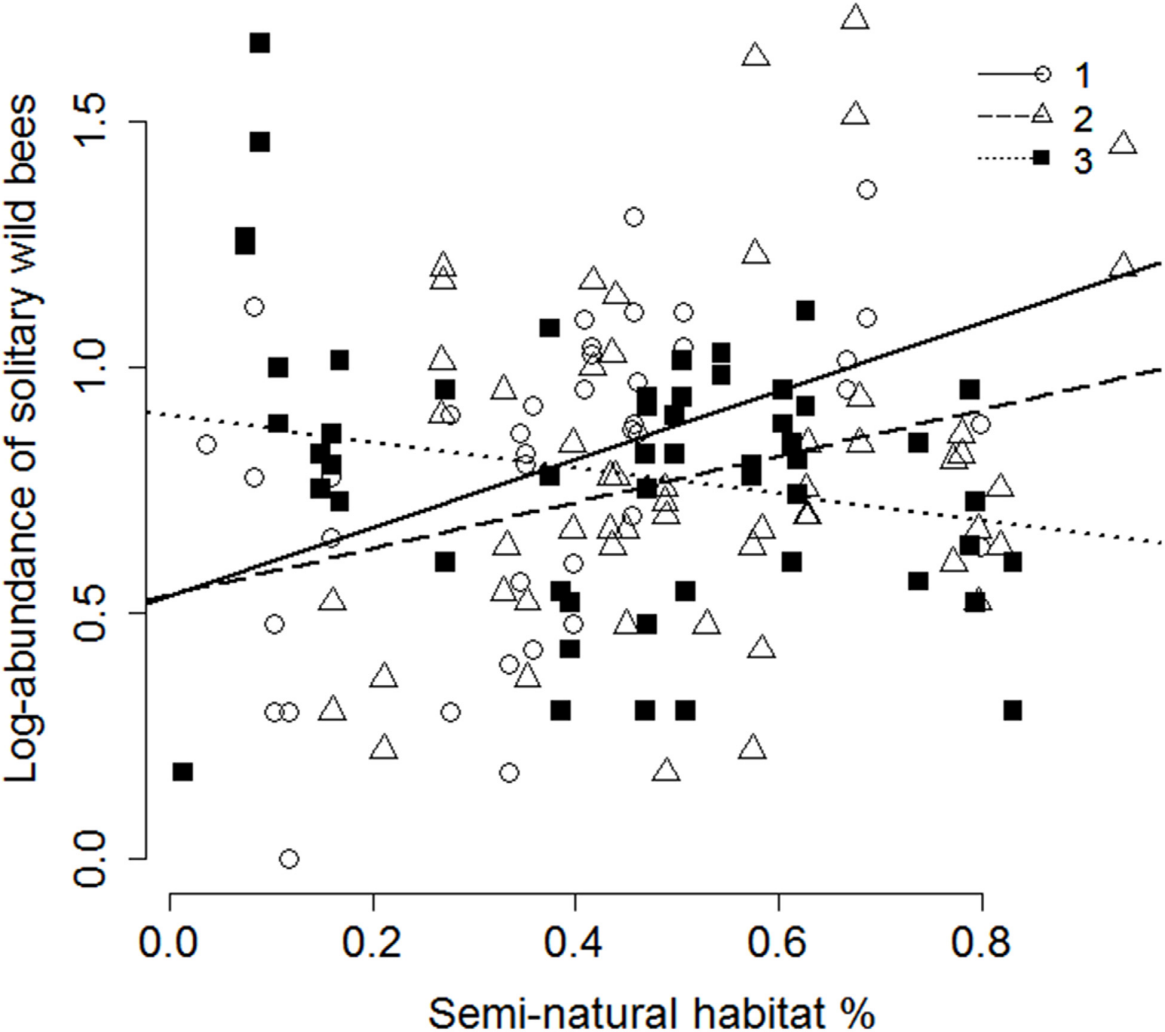
The effect of percentage of semi-natural habitats within 1000 m on the abundance of solitary wild bees as a function of increasing heterogeneity in a 1 ha radius circle around the studied fields. (Heterogeneity: 1 –low: Intercept = 0.536, Slope = 0.692, t = 3.13, p = 0.003; 2 –medium: Intercept = 0.537, Slope = 0.472, t = 2.09, p = 0.041, 3 –high: Intercept = 0.902, Slope = -0.266, t = -1.56, p = 0.124).

**Fig 5:**
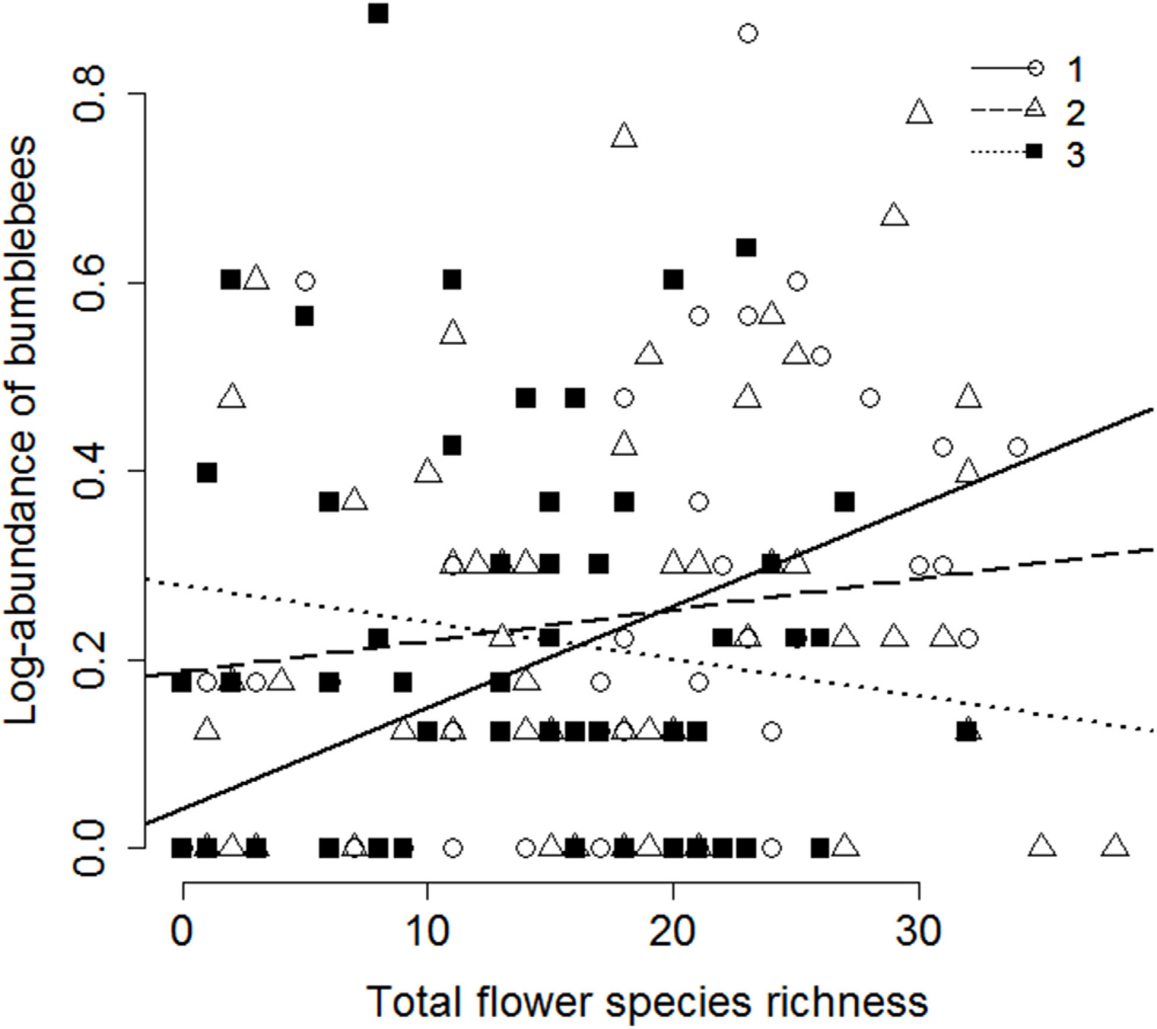
The effect of species richness of flowering insect-pollinated plants on the abundance of bumblebees as a function of increasing heterogeneity in a 1 ha radius circle around the studied fields. (Heterogeneity: 1 –low: Intercept = 0.042, Slope = 0.012, t = 3.24, p = 0.002; 2 –medium: Intercept = 0.187, Slope = 0.003, t = 1.12, p = 0.269, 3 –high: Intercept = 0.278, Slope = -0.004, t = -1.04, p = 0.301).

Significant interaction was found between percentage of semi-natural habitats within 1000 m and heterogeneity in 1ha in the case of wild bee and hoverfly species richness, and solitary wild bee abundance. In the case of wild bees and solitary wild bees semi-natural habitats within 1000 m had significant effect on pollinators in the sites of low heterogeneity within 1ha, but had no effect in the case of high heterogeneity (Fig 4). In the case of hoverflies, no semi-natural habitat % effect was found in the sites of low and medium heterogeneity, but there was a significant negative effect in the case of high heterogeneity. Heterogeneity and woody vegetation cover within 1 ha interacted significantly in the case of solitary wild bee abundance. There was significant interaction effect of heterogeneity in 1 ha and local flower species richness on the abundance of bumblebees: flower species richness had significant effect in the case of low heterogeneity, but no effect at medium or high heterogeneity values (Table 2; Fig 5).

The species composition of wild bees was significantly influenced by the percentage of semi-natural habitats and the Shannon land cover diversity within 1000 m, and flower species richness. Species composition of hoverflies was only influenced by the percentage of semi-natural habitats within 1000 m (Table 3).

**Table 3:** The effects of percentage of semi-natural habitats and Shannon land-cover diversity in 1000 m radius, heterogeneity and woody vegetation cover in a given 1 ha circle area, and flower species richness on the species composition of wild bees and hoverflies according to the partial redundancy analyses. Significant effects (p < 0.05) are in bold.

	Wild bees		Hoverflies	
	Pseudo-F	p	Pseudo-F	p
Semi-natural %—1000 m	2.22	**0.001**	1.83	**0.028**
SHDI—1000 m	2.38	**0.001**	1.30	0.184
Heterogeneity—1 ha	1.04	0.395	1.14	0.306
Wood cover—1 ha	1.29	0.112	1.36	0.143
Flower species richness	3.44	**0.001**	1.70	0.059

Flower species richness was significantly influenced by crop (df = 6, F = 25.37, p < 0.001; S3 Fig).

## Discussion

### Drivers of pollinator communities in the studied traditional agricultural landscape

Our study focused on the effects of various landscape and local conditions on the pollinator communities in arable fields and grasslands, and provides hypotheses for predicting changes due to potentially altering management in this sustainably used environment, which is still rather diverse, but understudied. Our results suggest that (i) both landscape scale effects and local foraging resources have a considerable effect on pollinator insects, which could suffer from both land use and land management change in the studied traditional Transylvanian agricultural systems; (ii) both arable fields and grasslands are important to sustain pollinator communities with different manner in the case of the different pollinator groups; (iii) the currently non-protected areas can be important conservation areas for the studied pollinator insects; (iv) species richness and abundance of wild bee species of European scale conservation interest are not directly influenced by the studied landscape and local scale environmental variables, however represent new habitat use records in some cases.

#### Landscape and local scale drivers

Landscape composition and configuration can have an effect on different pollinator groups on a different way and at different scale, depending on their mobility, nesting and foraging needs [29]. We found that percentage of semi-natural habitats within 1000 m radius around the study sites had a positive effect on the polylectic wild bees. Higher proportion of the semi-natural habitats might correspond with higher species richness of available flower resources within the landscape [56], which enables higher species richness and abundance of polylectic bees, utilising a wider spectrum of flowering plants [57,58]. Besides semi-natural habitats such as grasslands are likely to contain a high density of wild bee, and in particular bumblebee nests [57]. Similarly the positive landscape scale semi-natural habitat percentage effect on the abundance of butterflies suggests higher amount of foraging resources for both the adults and the caterpillars [57]. Moreover, semi-natural grasslands act as population sources from which individuals disperse to the surrounding habitats and thereby contribute to higher densities and species richness in adjacent areas [57]. In contrast negative correlation with hoverfly abundance reflects the importance of arable fields for hoverflies that could be important overwintering and foraging habitat especially for the aphidophagous species, ensuring also efficient aphid predation and important biological control [59]. Furthermore the wind pollinated crops in arable fields provide more pollen, which is essential food resource for the imagoes during breeding and egg laying period, therefore these areas are used as habitat by hoverflies in these lifestages [60]. However, semi-natural habitats can play also important role for hoverflies [61], as their density strongly depends on resource quantity, such as the amount of pollen and nectar resources for adults and the amount of larval macrohabitats [62].

Percentage of semi-natural habitats interacted significantly with heterogeneity of the closer surroundings around the fields in the case of species richness of wild bees and abundance of solitary wild bees. Importance of landscape context for the species richness of flower visiting insects depends upon the quality of the habitat patches [63]. Solitary bees have mostly more limited foraging range and therefore being less exposed to landscape scale characteristics further than few hundred meters from their nest [39,64,65]. However, if flower resources are limited close to their nests, they tend to forage at higher distances [39]. Therefore higher percentage of semi-natural habitats within 1000 m and potentially higher amount of associated flowering plants can become more important if the closer environment is less heterogeneous and flower rich [63].

Bumblebees, however, showed higher abundance with increasing species richness of flowering plants in the fields of homogeneous, but not such tendency in the fields of heterogeneous structure within the field and in the vicinity. Landscape scale characteristics usually have stronger effects on bumblebees, because they are able to disperse several kilometres from their nest, if no closer food resources are available [66, 67]. Our results suggest that bumblebees are distributed among the various available foraging resources in more heterogeneous landscapes within 1 ha area, while in the case of lower heterogeneity, they are more concentrated and appear in higher abundance in habitats with increased flowering plant diversity.

In contrast hoverflies responded to the higher amount of semi-natural habitats with lower species richness in sites with high heterogeneity. Hoverfly species richness is influenced by resource heterogeneity such as species richness of flowering plants, area of grassland habitat, and landscape diversity, which all imply the availability of diverse micro- and macrohabitats for adults and larvae [62]. Hoverflies have strong dispersal abilities and are highly mobile and they can therefore easily settle in new habitats [58]. In Transylvania there are many semi-natural habitats, pastures, woody vegetation in agricultural landscapes (habitat patches), which may increase the diversity of hoverflies and decrease the effect of local conditions [63].

#### Arable fields and grasslands for pollinator conservation

We found more than one-fifth of the Romanian wild bee species [68] and one-tenth of the Romanian hoverfly species [69] in our study that presents a rich pollinator community of the studied traditional agricultural landscape. Arable fields and grasslands both comprised high number of species and individuals of pollinators, with significant differences among the different crop and management types. One of the main drivers of pollinator occurrence is availability of flower resources [56]. The survival of bees, hoverflies and butterflies strongly depends on the availability of food resources for imagoes and larvae [52,53]. The quantity and quality of pollen and nectar sources of flowering plants are important factors in overwintering, mating or egg maturation of imagoes [58,62]. Flower species richness had strong associations with land use type and crop/management of the studied fields, showing higher values in grassland compared to arable fields. Especially shrubby grasslands, hay meadows, fallows, pastures were flower rich, while less flower species were found in alfalfa and cereal fields, and very few in corn.

The higher species richness of wild bees, especially those of polylectic species in grasslands corresponds with the wider range of available flower species there, although they showed high values in the flower rich fallows as well. The higher abundance of bumblebees and oligolectic wild bees in the arable fields is mainly determined by the alfalfa sites and fallows. Alfalfa provided high amount of flowers, which were well utilised by bumblebees (especially *Bombus terrestris*) and some oligolectic wild bee species such as *Andrena labialis*, *Eucera nigrescens*, *Melitta leporina*.

Hoverflies showed high abundance in alfalfa and fallows, but similar values were found also in cereal fields. Beside pollen consumption several species forage on aphids (Hemiptera: Aphidoidea), which make hoverflies important biological control agents in cereal fields [70]. *Sphaerophoria* spp. occurred in the highest numbers in cereal fields, alfalfa and fallows, which are known as important aphidophagous species [71].

The significant higher abundance of butterflies in grasslands compared to arable fields is in line with several European studies, considering grasslands as the most important habitats for butterfly conservation [72], but this result is in contrast with a parallel study on butterflies partly on the same sites by Loos et al. [31]. It might be due to the lower number of study sites and the less intense pollinator survey in our study. In general variables of the local habitat quality such as nectar plant abundance is suggested to have the highest effect on species richness and total density of butterflies and moths [73], therefore the more flower rich grasslands in our study area can support higher abundance of butterflies.

#### Transylvanian wild bee species of conservation interest at European level and conservation areas for wild bee species

We found three individuals of one species classified as endangered in Europe according to the IUCN Red bee list [45], *Halictus semitectus*. All three were found at the same alfalfa field of high heterogeneity and medium woody cover landscape. *H*. *semitectus* is a rare and steppic species, its area of occupancy is 108 km² of severely fragmented distribution, and there is still a continuing decline in the number of mature individuals. The species is known to inhabit deciduous forests (Poland) and steppe habitats [74], while our records are from an arable field. We found seven individuals of one of the vulnerable species, *Halictus leucaheneus* in the same grassland area of high heterogeneity and woody cover, while one individual of the other vulnerable species *Systropha planidens* was found in a cereal field. These records suggest the importance of maintaining not only diverse landscape structure, but also traditional arable fields for the conservation of wild bee species. The presence of these endangered, threatened, and vulnerable wild bee species in these fields and grasslands highlights the importance of traditional Transylvanian landscapes for their conservation at the regional scale, which in turn is important for the conservation of biodiversity at European scale. Furthermore areas that have no protection status (neither SCI or SPA) regard considerable interest, since several pollinator groups showed higher species richness and/or abundance according to our results.

### Implications for potential change due to EU accession

Changing land use and farmland management have strong effects on pollinator communities, which might face new threats in the traditional Romanian landscapes, and other extensively managed regions of CEE countries, due to the altering production demands, agricultural practices and subsidy systems through their EU accession. Both ceasing and intensifying management leads to alterations in vegetation and the related insect communities [15]. Land abandonment is more probable at the higher elevations, where remote fields in small valleys are usually less accessible, and non-profitable [27,75], while larger and more open fields of lower topography might be more likely to undergo intensification and loss of semi-natural habitats. Examples of both scenarios exist, e.g. Portugal and Spain, where huge areas of traditionally managed landscapes were degraded during the 70’s and 80’s [76]. Such land use and management changes could endanger the specific wild bee, hoverfly and butterfly communities and lead to decreasing pollinator diversity at regional level [15]. Agricultural management prescriptions can be therefore scale dependent even at regional level, not only at western-eastern European scale.

Comparing cropland and grassland management of the studied Transylvanian region to western Europe, inorganic fertiliser use in croplands is still very low in the study regions, reaching only 50–90 kg/ha (or even 0 kg/ha at some of our study sites) nutrient content in 2010 [28], which is comparable with allowed nitrogen per hectare in e.g. UK agri-environmental schemes [77] or organic fields in Germany [56], while average nutrient content of used inorganic fertiliser in conventional fields can reach even 300 kg/ha in arable fields and 100 kg/ha in grasslands in the UK [24] and 200 kg/ha in arable fields in Germany [56]. In contrast, organic fertiliser use is high in the studied traditional landscapes, however, it shows decreasing tendency in the most intensified Mureş and Harghita counties (Fig 2).

Grassland management is also changing in the region. Grazing by sheep and cattle declined in the study area after the collapse of the socialist system, but the number of sheep has started to increase again in the last 10–15 years, reaching 125–200 sheep / 100 ha in 2013 (Fig 3), which is more than one magnitude lower than grazing intensity in UK pastures [24].

Intensified agricultural land use with an increased use of artificial fertiliser and herbicide has been shown to impoverish arable and grassland plant communities [78], and to reduce pollinator food resources [10], just as it has been shown for intensive grazing or mowing in grasslands [9]. The different life cycle of the different species results in continuous presence of pollinators and ensures suitable pollination of actually flowering plants, but from the pollinators’ perspective also pose crucial requirement for continuous flower resources [79–81]. This natural pattern is largely missing from Western European countries, where targeted agri-environmental schemes were introduced to establish sown margins of different flower mixtures to provide foraging resources for wild pollinators and attract them to the crop fields, enhancing pollination efficiency [82,83]. Maintenance of the mosaic crop structure, smaller fields and semi-natural habitats in the agricultural landscapes of our study area and other parts of CEE, however, would make such artificial solutions unnecessary.

On the other hand, cessation of management, such as grazing, mowing on grasslands, leads to vegetation succession that can have considerable negative consequences on the pollinator fauna [84]. Therefore, maintenance of optimal management at such valuable, traditionally managed systems is highly recommended.

## Conclusions

In the less intensively managed agricultural landscapes of Central and Eastern Europe, which still harbour valuable biodiversity, maintenance of traditional farming landscapes has considerable conservation policy relevance [12]. Our study provides a baseline of how pollinator communities are organised under low-intensity traditional agriculture in Transylvania. Several of our results have important implications for farmland management: (i) the positive effects of flower diversity and abundance on pollinator communities seem to be a general pattern across European farmlands, however, flower resources should be continuously available, which calls for more diverse management. (ii) high proportions of semi-natural vegetation in landscapes play a crucial role as foraging and nesting habitats for pollinators, as it was shown already in several Western European studies. However, within traditional farmlands in addition semi-natural habitats, the arable fields are also suitable for pollinator insects and harbour a distinct and rich pollinator community due their rich weedy flora. This challenges the expectation that a few meter wide or other small areas of flower resources are suitable for the conservation of pollinators in intensive farmlands.

Due to lack of evidence, it is hard to predict how pollinator communities might change in the studied Transylvanian traditional landscapes as a result of the potential changes posed by the recent EU accession. Based on our results we pose the following general hypotheses, which could highlight the potential threats of EU accession and indicate new research initiatives. We hypothesise that although several management practices are similar in our traditional study areas and in fields in old EU countries with AESs, the corresponding biodiversity values are largely different, with much higher values including rare species in the traditional fields. It may indicate that AESs are far from reaching the baseline in conservation status. This hypothesis needs further study, which includes more traditional landscapes providing baseline, and exploration of other factors behind the lower than baseline level biodiversity values of AES fields.

Our study demonstrates the importance of deliberate, knowledge-based actions in future policy decisions in Romania to maintain traditional agricultural landscapes. However, traditional farming needs traditional farmers. The most efficient way to maintain these traditional land uses if well-being of local people can be enhanced to a level acceptable for them [85]. Therefore there is an urgent need for policies that foster a wider approach to socioeconomic development but also protect biodiversity [26]. High public awareness of ecological values and strong institutions dealing with environmental issues at both government and civil society level are needed [25].

## Supporting Information

S1 Table Explanatory variables applied in the general linear mixed effect models. Minimum (Min), maximum (Max), arithmetic mean (Mean) and standard deviation (SD) are listed.
(DOCX)

S2 Table Species list of wild bees with their abundance (number of individuals) in arable fields and grasslands, lecty category and conservation interest in the studied arable fields and grasslands in Transylvania, Romania. IUCN European Red bee list categories are presented at geographical Europe scale (EN—Endangered; VU—Vulnerable; NT—Near Threatened; LC—Least Concern; DD—Data Deficient).
(DOCX)

S3 Table Species list of hoverflies with their abundance (number of individuals) in the studied arable fields and semi-natural grasslands in Transylvania, Romania. (DOCX)

S4 Table The effect of crop on the species richness and abundance of the different pollinator groups according to the ANOVA. (DOCX)

S1 Fig Google Earth map of the studied arable fields and grasslands. Sites were assigned in the vicinity of 19 villages in the Transylvanian Basin, Romania. Sites are named by the first three letters of the closest village, A (arable) or P (grassland) indicates the land use type, plus H (heterogeneity) and W (woody vegetation cover) values in a 3 value range. Heterogeneity was defined as the standard deviation of the 2.5m panchromatic Spot picture, stratified in quantiles: H1 –belonging to lower third (low heterogeneity), H2 –middle third, H3 –upper third. Woody vegetation cover represented the proportion of woody vegetation (shrubs and trees) in a 1 ha circle based on classified 10m SPOT 5 data: W1: 0 to 5% woody vegetation cover; W2: 5 to 15% of woody vegetation; W3: 15 to 50% of woody vegetation (1 –low, 2 –middle, 3 –high).
(KML)

S2 Fig Species richness of a) wild bees, b) oligolectic wild bees, c) polylectic wild bees, d) wild bees of conservation interest, e) hoverflies, and abundance of f) solitary wild bees, g) bumblebees, h) oligolectic wild bees, i) polylectic wild bees, j) wild bees of conservation interest, k) hoverflies, l) butterflies in the function of the different crop types (mean ± 95% confidence interval). Non-overlapping confidence intervals represent significant difference between the crop types.
(DOCX)

S3 Fig Species richness of flowering plants in the function of the different crop types (mean ± 95% confidence interval). Non-overlapping confidence intervals represent significant difference between the crop types.
(DOCX)
