# Supplementary material for: Conservation of Pollinators in Traditional Agricultural Landscapes – New Challenges in Transylvania (Romania) Posed by EU Accession and Recommendations for Future Research
Source: PLoS One. 2016 Jun 10;11(6):e0151650. doi: 10.1371/journal.pone.0151650 (PMC4902286; doi:10.1371/journal.pone.0151650)
Supplement: S1 Table — Minimum (Min), maximum (Max), arithmetic mean (Mean) and standard deviation (SD) are listed. (DOCX) [file pone.0151650.s001.docx]

**S1 Table.** Explanatory variables applied in the general linear mixed effect models. Minimum (Min), maximum (Max), arithmetic mean (Mean) and standard deviation (SD) are listed.

|  |  |  |  |  |
| --- | --- | --- | --- | --- |
|  | Min | Max | Mean | SD |
| Semi-natural % - 1000 m | 0.01 | 0.94 | 0.45 | 0.22 |
| SHDI - 1000 m | 0.27 | 1.29 | 0.85 | 0.24 |
| Heterogeneity - 1 ha | 1.00 | 3.00 | 2.08 | 0.79 |
| Woody vegetation cover - 1 ha | 1.00 | 3.00 | 2.00 | 0.80 |
| Flower species richness | 0.00 | 38.00 | 16.31 | 9.02 |
|  |  |  |  |  |
