# Supplementary material for: Conservation of Pollinators in Traditional Agricultural Landscapes – New Challenges in Transylvania (Romania) Posed by EU Accession and Recommendations for Future Research
Source: PLoS One. 2016 Jun 10;11(6):e0151650. doi: 10.1371/journal.pone.0151650 (PMC4902286; doi:10.1371/journal.pone.0151650)
Supplement: S2 Table — IUCN European Red bee list categories are presented at geographical Europe scale (EN—Endangered; VU—Vulnerable; NT—Near Threatened; LC—Least Concern; DD—Data Deficient). (DOCX) [file pone.0151650.s002.docx]

**S2_Table.** Species list of wild bees with their abundance (number of individuals) in arable fields and grasslands, lecty category and conservation interest in the studied arable fields and grasslands in Transylvania, Romania. IUCN European Red bee list categories are presented at geographical Europe scale (EN – Endangered; VU – Vulnerable; NT – Near Threatened; LC – Least Concern; DD – Data Deficient).

|  |  |  |  |  |
| --- | --- | --- | --- | --- |
| **Species name** | **Arable** | **Grassland** | **Lecty** | **IUCN-Europe** |
| *Andrena aeneiventris* | 2 |  | Polylectic | LC |
| *Andrena bicolor* | 2 | 1 | Polylectic | LC |
| *Andrena dorsata* |  | 2 | Polylectic | DD |
| *Andrena flavipes* | 55 | 34 | Polylectic | LC |
| *Andrena fulvago* | 3 |  | Oligolectic | DD |
| *Andrena gelriae* | 1 | 3 | Oligolectic | DD |
| *Andrena hattorfiana* | 1 | 1 | Oligolectic | NT |
| *Andrena humilis* | 1 | 1 | Oligolectic | DD |
| *Andrena labiata* | 2 | 5 | Polylectic | DD |
| *Andrena labialis* | 41 | 8 | Oligolectic | DD |
| *Andrena limata* | 1 |  | Polylectic | DD |
| *Andrena minutula* |  | 5 | Polylectic | DD |
| *Andrena minutuloides* | 1 | 4 | Polylectic | DD |
| *Andrena nitidiuscula* | 15 | 3 | Oligolectic | LC |
| *Andrena nitida* | 1 |  | Polylectic | LC |
| *Andrena ovatula* | 29 | 54 | Polylectic | NT |
| *Andrena pallitarsis* | 1 | 1 | Oligolectic | DD |
| *Andrena pandellei* |  | 2 | Oligolectic | LC |
| *Andrena polita* |  | 3 | Oligolectic | LC |
| *Andrena rosae* |  | 1 | Oligolectic | DD |
| *Andrena schencki* | 1 |  | Polylectic | DD |
| *Andrena subopaca* | 3 | 10 | Polylectic | LC |
| *Andrena thoracica* |  | 1 | Polylectic | DD |
| *Andrena ventricosa* | 1 |  | Polylectic | DD |
| *Andrena viridescens* |  | 5 | Oligolectic | DD |
| *Andrena wilkella* | 5 |  | Oligo- or polylectic | DD |
| *Anthophora crinipes* |  | 3 | No Lectic status | DD |
| *Anthophora furcata* | 1 |  | Oligolectic | LC |
| *Anthophora plagiata* | 1 |  | Polylectic | LC |
| *Anthophora plumipes* | 2 |  | Polylectic | LC |
| *Anthophora pubescens* | 3 |  | Polylectic | DD |
| *Anthidium punctatum* | 1 |  | Polylectic | LC |
|  |  |  |  |  |

**S2_Table.** Continued.

|  |  |  |  |  |
| --- | --- | --- | --- | --- |
| **Species name** | **Arable** | **Grassland** | **Lecty** | **IUCN-Europe** |
| *Bombus hortorum* | 2 |  | Polylectic | LC |
| *Bombus humilis* | 31 | 15 | Polylectic | LC |
| *Bombus pascuorum* | 7 | 5 | Polylectic | LC |
| *Bombus pratorum* | 3 | 2 | Polylectic | LC |
| *Bombus ruderarius* | 1 | 6 | Polylectic | LC |
| *Bombus sylvarum* | 28 | 12 | Polylectic | LC |
| *Bombus terrestris* | 106 | 66 | Polylectic | LC |
| *Ceratina cyanea* | 1 |  | Polylectic | LC |
| *Ceratina nigrolabiata* | 1 | 2 | Polylectic | LC |
| *Chelostoma florisomne* |  | 2 | Oligolectic | LC |
| *Coelioxys afra* | 1 | 1 | No Lectic status | LC |
| *Coelioxys mandibularis* |  | 1 | No Lectic status | LC |
| *Colletes daviesanus* | 1 | 4 | Oligolectic | LC |
| *Colletes hylaeiformis* |  | 1 | Oligolectic | LC |
| *Colletes similis* |  | 5 | Oligolectic | LC |
| *Epeoloides coecutiens* |  | 1 | No Lectic status | LC |
| *Epeolus variegatus* |  | 1 | No Lectic status | LC |
| *Eucera chrysopyga* | 8 | 1 | Oligolectic | LC |
| *Eucera clypeata* | 7 | 3 | Oligolectic | LC |
| *Eucera interrupta* | 3 | 4 | Oligolectic | LC |
| *Eucera longicornis* | 9 | 2 | Oligolectic | LC |
| *Eucera nigrescens* | 38 | 10 | Oligolectic | LC |
| *Halictus confusus* | 3 | 9 | Polylectic | LC |
| *Halictus eurygnathus* | 33 | 51 | Polylectic | DD |
| *Halictus gavarnicus* | 70 | 181 | Polylectic | LC |
| *Halictus kessleri* | 10 | 7 | Polylectic | LC |
| *Halictus langobardicus* |  | 1 | No Lectic status | LC |
| *Halictus leucaheneus* |  | 7 | Polylectic | VU |
| *Halictus maculatus* | 8 | 31 | Polylectic | LC |
| *Halictus patellatus* |  | 1 | Polylectic | LC |
| *Halictus rubicundus* | 5 | 3 | Polylectic | LC |
| *Halictus scabiosae* | 3 | 1 | Polylectic | LC |
| *Halictus semitectus* | 3 |  | Polylectic | EN |
| *Halictus sexcinctus* | 2 | 6 | Polylectic | LC |
| *Halictus simplex* | 45 | 66 | Polylectic | LC |
| *Halictus smaragdulus* | 2 | 9 | Polylectic | LC |
| *Halictus subauratus* | 25 | 28 | Polylectic | LC |
| *Heriades crenulatus* |  | 1 | Oligolectic | LC |
| *Hoplitis leucomelana* | 1 | 5 | Polylectic | LC |
|  |  |  |  |  |

**S2_Table.** Continued.

|  |  |  |  |  |
| --- | --- | --- | --- | --- |
| **Species name** | **Arable** | **Grassland** | **Lecty** | **IUCN-Europe** |
| *Hylaeus angustatus* |  | 1 | Polylectic | LC |
| *Hylaeus annularis* | 2 | 1 | Polylectic | DD |
| *Hylaeus brevicornis* | 4 | 2 | Polylectic | LC |
| *Hylaeus communis* |  | 2 | Polylectic | LC |
| *Hylaeus confusus* | 3 |  | Polylectic | LC |
| *Hylaeus cornutus* |  | 1 | Polylectic | LC |
| *Hylaeus duckei* |  | 1 | Polylectic | DD |
| *Hylaeus sinuatus* | 1 | 2 | Polylectic | LC |
| *Hylaeus variegatus* |  | 2 | Polylectic | LC |
| *Lasioglossum albipes* | 1 | 9 | Polylectic | LC |
| *Lasioglossum brevicorne* |  | 4 | Oligo- or polylectic | NT |
| *Lasioglossum calceatum* | 10 | 15 | Polylectic | LC |
| *Lasioglossum corvinum* | 1 |  | Polylectic | LC |
| *Lasioglossum costulatum* |  | 2 | Oligolectic | NT |
| *Lasioglossum discum* | 2 | 20 | Polylectic | LC |
| *Lasioglossum fulvicorne* | 1 | 9 | Polylectic | LC |
| *Lasioglossum glabriusculum* | 30 | 105 | Polylectic | LC |
| *Lasioglossum griseolum* |  | 4 | Polylectic | LC |
| *Lasioglossum interruptum* | 49 | 50 | Polylectic | LC |
| *Lasioglossum laevigatum* | 1 |  | Polylectic | NT |
| *Lasioglossum laticeps* | 3 | 1 | Polylectic | LC |
| *Lasioglossum lativentre* | 5 | 2 | Polylectic | LC |
| *Lasioglossum leucozonium* | 5 | 3 | Polylectic | LC |
| *Lasioglossum lineare* | 2 | 6 | Polylectic | DD |
| *Lasioglossum lucidulum* | 3 |  | Polylectic | LC |
| *Lasioglossum majus* | 4 | 2 | Polylectic | NT |
| *Lasioglossum malachurum* | 119 | 50 | Polylectic | LC |
| *Lasioglossum marginatum* | 1 | 5 | Polylectic | LC |
| *Lasioglossum morio* | 19 | 21 | Polylectic | LC |
| *Lasioglossum nigripes* | 2 | 5 | Polylectic | LC |
| *Lasioglossum pauxillum* | 63 | 77 | Polylectic | LC |
| *Lasioglossum politum* | 19 | 14 | Polylectic | LC |
| *Lasioglossum punctatissimum* | | 4 | Polylectic | LC |
| *Lasioglossum puncticolle* | 2 |  | Polylectic | LC |
| *Lasioglossum truncaticolle* | 1 |  | Polylectic | DD |
| *Lasioglossum villosulum* | 3 | 18 | Polylectic | LC |
| *Lasioglossum xanthopus* |  | 3 | Polylectic | NT |
| *Lasioglossum zonulum* | 14 | 2 | Polylectic | LC |
|  |  |  |  |  |

**S2_Table.** Continued.

|  |  |  |  |  |
| --- | --- | --- | --- | --- |
| **Species name** | **Arable** | **Grassland** | **Lecty** | **IUCN-Europe** |
| *Macropis europaea* | 1 |  | Oligolectic | LC |
| *Megachile centuncularis* | 1 | 1 | Polylectic | LC |
| *Megachile ericetorum* | 1 | 1 | Oligolectic | LC |
| *Megachile melanopyga* |  | 1 | Oligolectic | LC |
| *Megachile pilidens* | 10 | 13 | Polylectic | LC |
| *Megachile rotundata* | 2 | 3 | Polylectic | DD |
| *Melitturga clavicornis* | 7 |  | Oligolectic | NT |
| *Melitta dimidiata* |  | 1 | Oligolectic | NT |
| *Melitta leporina* | 44 | 4 | Oligolectic | LC |
| *Melitta nigricans* | 2 |  | Monolectic | LC |
| *Melitta tricincta* | 1 |  | Oligolectic | NT |
| *Nomada alboguttata* |  | 1 | No Lectic status | LC |
| *Nomada basalis* |  | 2 | No Lectic status | LC |
| *Nomada bluethgeni* |  | 1 | No Lectic status | LC |
| *Nomada fucata* |  | 2 | No Lectic status | LC |
| *Nomada pleurosticta* |  | 1 | No Lectic status | NT |
| *Nomada trispinosa* | 1 |  | No Lectic status | LC |
| *Osmia aurulenta* | 2 | 2 | Polylectic | LC |
| *Osmia bidentata* |  | 1 | Oligolectic | LC |
| *Osmia cerinthidis* | 1 |  | Oligolectic | LC |
| *Osmia leaiana* |  | 1 | Oligolectic | LC |
| *Osmia rufohirta* | 1 | 6 | Polylectic | LC |
| *Osmia spinulosa* | 1 | 4 | Oligolectic | LC |
| *Osmia tergestensis* |  | 2 | Oligolectic | LC |
| *Panurgus calcaratus* |  | 2 | Oligolectic | LC |
| *Pasites maculatus* |  | 3 | No Lectic status | LC |
| *Pseudapis bispinosa* |  | 2 | Polylectic | LC |
| *Pseudapis diversipes* | 1 | 11 | Polylectic | LC |
| *Sphecodes ephippius* |  | 1 | No Lectic status | LC |
| *Sphecodes gibbus* |  | 7 | No Lectic status | LC |
| *Sphecodes monilicornis* | 1 |  | No Lectic status | LC |
| *Sphecodes reticulatus* |  | 2 | No Lectic status | LC |
| *Sphecodes rufiventris* | 1 | 3 | No Lectic status | LC |
| *Sphecodes scabricollis* |  | 1 | No Lectic status | DD |
| *Systropha curvicornis* | 13 | 1 | Oligolectic | NT |
| *Systropha planidens* | 1 |  | Oligolectic | VU |
| *Tetraloniella alticincta* | 6 | 3 | Oligolectic | LC |
| *Tetraloniella dentata* |  | 3 | Oligolectic | LC |
|  |  |  |  |  |

**S2_Table.** Continued.

|  |  |  |  |  |
| --- | --- | --- | --- | --- |
| **Species name** | **Arable** | **Grassland** | **Lecty** | **IUCN-Europe** |
| *Tetraloniella salicariae* | 1 | 1 | Oligolectic | DD |
| *Trachusa byssina* |  | 2 | Oligolectic | LC |
| *Triepeolus tristis* |  | 2 | No Lectic status | NT |
|  |  |  |  |  |
