# Supplementary material for: Conservation of Pollinators in Traditional Agricultural Landscapes – New Challenges in Transylvania (Romania) Posed by EU Accession and Recommendations for Future Research
Source: PLoS One. 2016 Jun 10;11(6):e0151650. doi: 10.1371/journal.pone.0151650 (PMC4902286; doi:10.1371/journal.pone.0151650)
Supplement: S3 Table — (DOCX) [file pone.0151650.s003.docx]

**S3_Table.** Species list of hoverflies with their abundance (number of individuals) in the studied arable fields and semi-natural grasslands in Transylvania, Romania.

| **Species** | **Arable** | **Grassland** |
| --- | --- | --- |
| *Cheilosia vulpina* | 1 |  |
| *Chrysotoxum bicinctum* | 8 | 5 |
| *Chrysotoxum cautum* | 2 | 19 |
| *Chrysotoxum elegans* | 1 | 2 |
| *Chrysotoxum festivum* | 6 | 11 |
| *Chrysotoxum lineare* | 1 |  |
| *Chrysotoxum vernale* | 2 | 5 |
| *Chrysotoxum verrali* | 1 |  |
| *Epistrophe diaphana* |  | 1 |
| *Epistrophe nitidicollis* | 3 |  |
| *Episyrphus balteatus* | 17 | 6 |
| *Eristalinus aeneus* | 2 | 7 |
| *Eristalis arbustorum* | 15 | 18 |
| *Eristalis tenax* | 12 | 12 |
| *Eumerus sogdianus* | 1 |  |
| *Eumerus strigatus* | 1 |  |
| *Eupeodes corollae* | 14 | 6 |
| *Eupeodes lapponicus* | 1 | 1 |
| *Eupeodes latifasciatus* | 1 |  |
| *Helophilus trivittatus* | 2 | 1 |
| *Lejogaster metallina* | 4 | 3 |
| *Mallota fuciformis* |  | 1 |
| *Melanogaster nuda* |  | 7 |
| *Melanostoma mellinum* | 12 | 2 |
| *Merodon equestris* |  | 1 |
| *Microdon analis* |  | 2 |
| *Microdon devius* | 2 | 1 |
| *Microdon miki* |  |  |
| *Microdon mutabilis* | 4 | 2 |
| *Myathropa florea* | 2 | 6 |
| *Paragus bicolor* |  | 1 |
| *Paragus haemorrhous* | 3 | 12 |
| *Paragus tibialis* |  | 1 |
| *Pipizella maculipennis* |  | 1 |
| *Pipizella viduata* | 2 | 14 |
| *Rhingia campestris* | 3 |  |

**S3_Table.** Continued

| **Species** | **Arable** | **Grassland** |
| --- | --- | --- |
| *Scaeva pyrastri* | 1 | 1 |
| *Sphaerophoria scripta* | 172 | 63 |
| *Sphaerophoria taeniata* | 31 | 6 |
| *Syritta pipiens* | 22 | 38 |
| *Syrphus ribesii* |  | 2 |
| *Syrphus torvus* |  | 1 |
| *Volucella bombylans* | 3 |  |
| *Xanthogramma pedissequum* | 3 | 4 |
