# Supplementary material for: Conservation of Pollinators in Traditional Agricultural Landscapes – New Challenges in Transylvania (Romania) Posed by EU Accession and Recommendations for Future Research
Source: PLoS One. 2016 Jun 10;11(6):e0151650. doi: 10.1371/journal.pone.0151650 (PMC4902286; doi:10.1371/journal.pone.0151650)
Supplement: S4 Table — (DOCX) [file pone.0151650.s004.docx]

**S4_Table**. The effect of crop on the species richness and abundance of the different pollinator groups according to the ANOVA.

|  |  |  |  |  |
| --- | --- | --- | --- | --- |
|  |  | **Crop** | | |
|  |  | df | F | p |
| **Species richness** | |  |  |  |
|  | Wild bees | 6 | 7.50 | **<0.001** |
|  | Oligolectic bees | 6 | 5.20 | **<0.001** |
|  | Polylectic bees | 6 | 10.23 | **<0.001** |
|  | Wild bees of conservation interest | 6 | 3.38 | **0.004** |
|  | Hoverflies | 6 | 2.69 | **0.017** |
| **Abundance** | |  |  |  |
|  | Solitary wild bees | 6 | 9.13 | **<0.001** |
|  | Bumblebees | 6 | 8.30 | **<0.001** |
|  | Oligolectic bees | 6 | 8.13 | **<0.001** |
|  | Polylectic bees | 6 | 6.97 | **<0.001** |
|  | Wild bees of conservation interest | 6 | 2.16 | **0.050** |
|  | Hoverflies | 6 | 5.18 | **<0.001** |
|  | Butterflies | 6 | 18.34 | **<0.001** |
|  |  |  |  |  |
