# Supplementary material for: Conservation of Pollinators in Traditional Agricultural Landscapes – New Challenges in Transylvania (Romania) Posed by EU Accession and Recommendations for Future Research
Source: PLoS One. 2016 Jun 10;11(6):e0151650. doi: 10.1371/journal.pone.0151650 (PMC4902286; doi:10.1371/journal.pone.0151650)
Supplement: S2 Fig — Non-overlapping confidence intervals represent significant difference between the crop types. (DOCX) [file pone.0151650.s006.docx]

**S1_Figure** Species richness of a) wild bees, b) oligolectic wild bees, c) polylectic wild bees, d) wild bees of conservation interest, e) hoverflies, and abundance of f) solitary wild bees, g) bumblebees, h) oligolectic wild bees, i) polylectic wild bees, j) wild bees of conservation interest, k) hoverflies, l) butterflies in the function of the different crop types (mean ± 95% confidence interval). Non-overlapping confidence intervals represent significant difference between the crop types.

a)

b)

c)

d)

e)

f)

g)

h)

i)

j)

k)

l)
