# Supplementary material for: Conservation of Pollinators in Traditional Agricultural Landscapes – New Challenges in Transylvania (Romania) Posed by EU Accession and Recommendations for Future Research
Source: PLoS One. 2016 Jun 10;11(6):e0151650. doi: 10.1371/journal.pone.0151650 (PMC4902286; doi:10.1371/journal.pone.0151650)
Supplement: S3 Fig — Non-overlapping confidence intervals represent significant difference between the crop types. (DOCX) [file pone.0151650.s007.docx]

**S2_Figure**. Species richness of flowering plants in the function of the different crop types (mean ± 95% confidence interval). Non-overlapping confidence intervals represent significant difference between the crop types.
